# Quality of life and its association with current substance use, medication non-adherence and clinical factors of people with schizophrenia in Southwest Ethiopia: a hospital-based cross-sectional study

**DOI:** 10.1186/s12955-020-01340-0

**Published:** 2020-03-30

**Authors:** Defaru Desalegn, Shimelis Girma, Tilahun Abdeta

**Affiliations:** 1 Department of Psychiatry, Faculty of health and medical sciences, Mettu University, Mettu, Ethiopia; 2 Department of Psychiatry, Institute of health, Jimma University, Jimma, Ethiopia; 3 Department of Psychiatry, college of health and medical sciences, School of Nursing and Midwifery, Haramaya University, Harar, Ethiopia

**Keywords:** Quality of life, Medication non-adherence, Current substance use, Clinical factors, Schizophrenia, Ethiopia

## Abstract

**Background:**

Schizophrenia was ranked as one of the top ten illnesses contributing to the global burden of disease. But little is known about the quality of life among people with schizophrenia, in particular in low-income countries. This study was aimed to examine the association of quality of life with current substance use, medication non-adherence and clinical factors of people with schizophrenia at Jimma University Medical Center, psychiatry clinic, Southwest Ethiopia.

**Methods:**

Institution based cross-sectional study design was employed. Study participants were recruited using a systematic random sampling method and a sample fraction of two was used after the first person was identified by the lottery method. we used the World Health Organization Quality of Life Scale-Brief version (WHOQoL-BREF) and 4-item Morisky Medication Adherence Scale (MMAS-4) to assess the quality of life and medication non-adherence respectively. Data about current substance use was assessed by yes/no questions. Descriptive statistics, such as frequency, mean and standard deviations were computed to describe the characteristics of the study population. Data entry was done using EpiData version 3.1 then exported to SPSS statistics version 25 for analysis and analyzed using multiple linear regression. The assumption for linear regression analysis including the presence of a linear relationship between the outcome and predictor variable, the test of normality, collinearity statistics, auto-correlation and homoscedasticity were checked. Un-standardized Beta (β) coefficients with 95% confidence interval (CI) and *P*-value < 0.05 were computed to assess the level of association and statistical significance in the final multiple linear regression analysis.

**Result:**

In this study 31.65% of participants were medication non-adherent and total mean scores of quality of life showed a lower level of satisfaction in social relationship domain (10.14 ± 3.12). Our study showed 152(43.3%), 248(70.7%) and 97(27.6%) of respondents had used tobacco, Khat and alcohol atleast once during the past 3 months respectively. Final adjusted multiple regression model showed medication non-adherence has significant negative association with physical domain (beta = − 4.42, *p* < 0.001), psychological (beta = − 4.49, p < 0.001), social relationships (beta = − 2.29, *p* < 0.001) and environmental domains (beta = − 4.95, p < 0.001). Treatment duration has significant negative association with psychological domain (beta = − 0.17, *p* < 0.04), social relationship (beta = − 0.14, *p* < 0.005), environmental domain (beta = − 0.24, *p* < 0.02) and overall quality of life (beta = − 0.67, p < 0.02). Having comorbid physical illness has significant negative association with physical domain (beta = − 2.74, *p* < 0.001), psychological (beta = − 2.13, *p* < 0.004), social relationships (beta = − 1.25, *p* < 0.007), environmental domain (beta = − 3.39, *p* < 0.001) and overall quality of life (beta = − 9.9, *p* < 0.001). Current tobacco use has significant negative association with physical domain (beta = − 1.16, *p* < 0.004), psychological (beta = − 1.23, *p* < 0.001), social relationships (beta = − 0.88, *p* < 0.001), environmental domains (beta = − 1.98, *p* < 0.001) and overall quality of life (beta = − 5.73, *p* < 0.001). Also, current chewing khat has significant negative association with physical domain (beta = − 1.15, *p* < 0.003), psychological (beta = − 1.58, p < 0.001), environmental domains (beta = − 2.63, p < 0.001) and overall quality of life (beta = − 6.22, p < 0.001).

**Conclusion:**

The social relationship domain of quality of life has the lowest mean score. Medication non-adherence, treatment duration, having a comorbid physical illness, current tobacco use and current chewing khat were found to have a statistically significant association with the overall quality of life. Therefore, treatments aimed to improve social deficits, medication non-adherence, comorbid physical illness and decrease substance abuse is imperative.

## Background

Schizophrenia is one of the most severe, chronic and disabling mental disorders [1]. It affects general health, functioning, autonomy, subjective wellbeing, and alters individuals’ perception of reality [2]. Globally, as estimated by the World Health Organization (WHO), about 24 million people suffer from schizophrenia [3]. It was ranked as one of the top ten illnesses contributing to the global burden of disease [4]. In developing countries, around 90% of people with schizophrenia remain untreated [5]. In Ethiopia, mental illness is the leading non-communicable disease in terms of burden. Indeed, in a predominantly rural area of Ethiopia, mental illness comprised 11% of the total burden of disease, with schizophrenia and depression included in the top ten most burdens [6].

Schizophrenia affects many areas of functioning. People with the illness often lead an isolated and marginalized existence in poor housing, with a low income, little education, and poor vocational and social skills [2]. Most individuals are employed at a lower level and the majority of them have limited social contacts outside of their family [7]. People with schizophrenia are also prone to stigma, which leads to discrimination and thus affects their life opportunities and quality of life (QoL) [8].

Quality of life in individuals with schizophrenia has been measured from both subjective and objective points of view. Subjective measures of QoL include general indicators of life satisfaction and several life domains such as satisfaction with work, family, social relations, finances, and housing situations. The objective measures of QoL usually include indicators of external life conditions, socio-demographic items, and a functioning role in society [9]. However, the World Health Organization (WHO) focused on the subjective aspect and defines the quality of life as individuals’ perception of their position in life in the context of the culture and value systems concerning their goals, expectations, standards, and concerns [10].

A study done in China found that people with schizophrenia who were treated in primary care had a lower level of QoL when compared to the general population [11]. A cross-sectional comparative study done in Pakistan showed that people with schizophrenia had significantly poorer quality of life when compared with healthy subjects [12] and also they have worse than that of other physically ill people [13]. A study conducted in Bangladesh found out, most of the schizophrenic patient lead poor to moderate quality of life in four domains of the World Health Organization Quality of Life Scale – Brief Version (WHOQOL-BREF scale) [14].

Quality of life of patients with schizophrenia can be affected by different factors including socio-demographic variables, type of psychopathology [15, 16], social support and substance use disorders [17]. A study done in Spain among people with schizophrenia found that young people, women, married persons, and those with a low level of education report better quality of life [13]. Also, a study from Jordan reveals that QoL of people with schizophrenia was correlated positively with social support, individuals’ educational, income level, and employment [16]. But, one study done in Sweden showed that the socio-demographic indicators have a weak influence on the patient’s self-assessed quality of life [18].

Several studies have evaluated the associations between quality of life and clinical factors among individuals with schizophrenia. As a study done in Jordan, QoL of people with schizophrenia was correlated negatively with the severity of psychiatric symptoms, duration of untreated illness and duration of treatment [16]. Thus, the longer the length of the illness is associated with the worse the quality of life of people with schizophrenia [13].

A one-year randomized clinical trial (RCT) study done in the United Kingdom (UK) among 363 people with schizophrenia has indicated as improved adherence to medication leads to improved QoL [19]. As a study done in Nigeria among 313 people with schizophrenia attending outpatient treatments, 40.3% of the respondents were medication non-adherent and respondents with poor medication adherence had lower scores on all domains of quality of life and the facets of Overall QoL and General Health compared with medication-adherent subjects [20]. Also, another study done in Nigeria found that there are significant negative correlations between the overall health satisfaction, physical and psychological domains of quality of life and medication adherence: i.e. participants with poorer medication adherence were more likely to have poorer mean scores on the overall QoL, health satisfaction, physical and psychological domains [21].

Another factor that affects the quality of life of patients with schizophrenia is substance use status. According to one study conducted in Turkey among patients dually diagnosed with schizophrenia and substance use disorders and in non-substance-using male schizophrenia outpatients, there are significantly lower QoL scores in the comorbid group, specifically in the psychological domain of WHOQOL-BREF [17]. Contrary to this, dual-diagnosed patients with schizophrenia and substance use in the study from Australia expressed higher levels of satisfaction with their QoL compared with non- comorbid patients [22].

Assessing the determinant factors of quality of life of people with schizophrenia is particularly important to minimize the impact of the disorder and to improve their quality of life. In Ethiopia, only limited national wide studies conducted on the quality of life and associated factors among people with schizophrenia and especially there was no study addressed the association of medication non-adherence and current substance use with quality of life and again no study conducted specifically in the study area. Therefore, this study aimed to examine the quality of life and its association with current substance use, medication non-adherence and clinical factors of people with schizophrenia and attending follow up service at Jimma University Medical Center, psychiatry clinic, Southwest Ethiopia.

## Materials and methods

### Study setting

We conducted this study from April 15–June 15, 2018, among people with schizophrenia and attending, follow up service at Jimma University Medical Center (JUMC), psychiatry clinic, Southwest Ethiopia. Jimma University Medical Center is one of the oldest governmental hospitals, established in 1937 during the Italian occupation for the service of their soldiers [23]. The psychiatric clinic of JUMC was established in 1988 and gives clinical service for about 15 million population with a total of 40 beds for in-patient service and seven rooms for Out Patient Department (OPD) in Southwest Ethiopia during the study time [23]. Currently, there are more than 1000 people with schizophrenia who are attending follow up treatments at OPD monthly and on average, about 50 individuals with different mental illnesses are visiting psychiatric clinics daily.

### Study design and participants

The hospital-based cross-sectional study design was conducted among people with schizophrenia and who were attending follow up service at Jimma University Medical Center, psychiatric clinic from April 15–June 15, 2018. Three hundred fifty-one adult persons (age 18 and above) who were diagnosed as schizophrenia according to the diagnostic criteria of the Diagnostic Statistical Manual (DSM-IV/V) by psychiatrists or by psychiatric nurses under the consultation of psychiatrists were included in the study. People whose acute episode symptoms pose difficulty to communicate were excluded from the study.

### Sample size determination and sampling technique

The minimum sample size required for this study was determined by using the formula to estimate single population mean; using the assumptions of margin of error (d) 1; standard deviation of mean quality of life score 9.13 as reported by previous related study [18]; Z value at (∞ = 0.05) to be 1.96 and adding 10% non-response rate. Thus, the total sample size was 352. Study participants were recruited by using a systematic random sampling method and a sample fraction of two was used after the first person was identified by using the lottery method. To avoid repeated inclusion of a patient into the study unique patient identification card number was used as a questionnaire code.

### Data collection instrument and technique

Data were collected by face to face interviews using semi-structured and pre-tested questionnaires. Data collection tools were prepared first in English and translated into local languages (Afaan Oromo and Amharic) then re-translated back to English by another person who was blinded for the English version to check the clarity of the questionnaire. Data collectors were four (4) Master of Science in integrated clinical and community mental health students. One day training on the study objectives, data collection methods, ethical issues, and data collection tools was given for data collectors and the supervisors. The pre-test was conducted on 5% of the sample. Data collectors were supervised by two Masters of Science students in public health and the principal investigator. On each day of the data collection, the completed questionnaires were checked for completeness and finally, the collected data were entered into a computer and processed.

The questionnaire has the following components: Socio-demographic factors (age, sex, ethnicity, religion, marital status, educational status, occupational status, and monthly income); current substance use (khat, smoking cigarettes and alcohol); medication non-adherence; clinical factors (duration of illness, duration of treatment, history of admission and comorbid physical illness) and World Health Organization Quality of Life Scale-Brief version (WHOQoL-BREF) to assess the quality of life.

WHOQoL-BREF is 26 items self-administered generic questionnaire and it is a short version of the WHO QoL-100 scale [24]. This tool is a sound, cross-culturally valid assessment of QoL [25] and it is found a suitable tool for the assessment of QoL of people with schizophrenia [26]. WHOQOL-BREF was found to have good reliability (a high internal consistency) and validity which grants suitability of the tool for the person with schizophrenia [25]. The tool has four domain scores: physical health (7 items), psychological health (6 items), social relationships (3 items), environmental health domain (8 items) as well as two separately scored items about the individuals’ perception of their quality of life (QI) and health (Q2). The scores of each item in each domain were added, in case of negative items (i.e. the response of Q3, Q4, and Q26 was reverse coded); and multiplied by 4 to be directly comparable with WHOQOL-100. Therefore, Domain scores are scaled in a positive direction (i.e. higher scores correspond to a better quality of life). The WHOQoL-BREF scale in this study demonstrated a high internal consistency reliability coefficient (Cronbach’s alpha = 0.96). Regarding medication non-adherence, it was measured by the 4-item Morisky Medication Adherence Scale (MMAS-4) [27]. It was a self-reported medication-taking behavior scale. It consists of four items with a scoring scheme of Yes = 1 and No = 0. The items in MMAS-4 were summed to give a range of scores from zero to four (0–4). For this study, participants who scored 1 or more were considered as non- adherent while those who scored 0 was taken as adherent. The Cronbach’s α in this study was 0.77. We collected data about current substance use by yes/no questions. Finally data about having the comorbid physical illness was recorded by reviewing their medical records.

### Data processing and analysis

Collected data were checked, coded and entered into EpiData Version 3.1 and then exported to Statistical Package for Social Science Version 25.0 for further analysis. Descriptive statistics, such as frequency, mean and standard deviations were computed to describe the characteristics of the study population. The assumption for linear regression analysis including the presence of a linear relationship between the outcome and predictor variable, the test of normality, collinearity statistics, auto-correlation and homoscedasticity were checked. Bivariate linear regression analysis was done for each independent variable against the dependent variable and variables with *p*-value < 0.25 were considered as a candidate for multiple linear regression. Finally, multiple linear regression analysis was done to identify factors associated with quality of life. In the final adjusted multiple linear regression, variables with a *P*-value < 0.05 were declared statistically significantly associated with quality of life. Un-standardized Beta (β) coefficients with 95% confidence interval (CI) were computed to assess the level of association and statistical significance in multiple linear regression analysis.

### Data quality control

The questionnaire used in this study was prepared first in English and translated into local languages (Afaan Oromo and Amharic) then re-translated back to English by another person who was blinded for the English version to check the clarity of questionnaire. In our study, the internal consistency reliability coefficient (Cronbach’s alpha) for the WHOQoL-BREF scale was 0.96 and for the 4-item Morisky Medication Adherence Scale (MMAS-4) was 0.77. One day training was given for data collectors and supervisors. The pre-test was conducted on 5% of the sample to identify potential problems on data collection tools and to check the consistency of the questionnaire and modification of the questionnaire. Regular supervision and support were given for data collectors by the supervisors and principal investigator to ensure that all necessary data are properly collected. The collected data were checked for completeness and consistency by supervisors and principal investigators on a daily bases during the data collection time.

### Operational definitions

#### Schizophrenia

It is a clinical diagnosis reached by the clinician based on DSM-IV/V diagnostic criteria [1].

#### Medication non-adherence

Any participant scoring more than 0 by Morisky Medication Adherence Scale (MMAS-4) was considered as non- adherent while those with 0 scores were taken as adherent [20].

#### Current substance use-

Using any psychoactive substances at least once during the past 3 months [28].

### Ethical consideration

Ethical clearance was obtained from the Research Ethical Review Board of Jimma University, Institute of health and the study was performed according to the declaration of Helsinki. Written informed consent was obtained from study participants and they were told the right to refuse or discontinue participation at any time they want and the chance to ask anything about the study. After data entry was completed, the questionnaires were kept securely locked.

## Results

### Socio-demographic, medication non-adherence, clinical factors and current substance use data of study participants

In this study, the participants’ mean age was 33 years (SD 8) with the minimum and maximum age 18 and 54 years respectively. More than half of the study participants were married and (*n* = 242, 68.9%) were males and (*n* = 203, 57.8%) were urban residents. Many of them (23.9%) were farmers followed by government workers (22.5%). Concerning their educational status, 28.2% of them attended primary school and followed by those who had no formal education (27.1%). Regarding their current substance use, 152(43.3%), 248(70.7%) and 97(27.6%) had used tobacco, Khat, and alcohol at least once during the past 3 months respectively. Regarding the medication non-adherence status of the participants, as measured by MMAS-4, of the 351 people with schizophrenia included in this study, 111(31.65%) were non-adherent to the medications (Table 1**)**.

**Table 1:** Socio-demographic, medication non-adherence, clinical factors and current substance use data of people with schizophrenia (*n* = 351)

Variables	Variable categories	Frequency(n)	Percentage(%)
Sex	Male	242	68.9
	Female	109	31.1
Marital status	Never married/single	120	34.2
	Married	193	55.0
	Divorced	30	8.5
	Widowed	8	2.3
Religion	Muslim	228	65.0
	Orthodox Christian	66	18.8
	Protestant Christian	57	16.2
Ethnicity	Oromo	236	67.2
	Amhara	38	10.8
	Yem	6	1.7
	Dawuro	13	3.7
	Kaffa	30	8.5
	Other^a^	28	8.0
Occupational status	Government worker	79	22.5
	Farmer	84	23.9
	Merchant	35	10.0
	Housewife	45	12.8
	Daily labor	45	12.8
	Students	63	17.9
Educational status	No formal education	95	27.1
	Primary school	99	28.2
	Secondary school	77	21.9
	Above secondary	80	22.8
Residence	Urban	203	57.8
	Rural	148	42.2
Age	<= 33	179	51.0
	> 33	172	49.0
Current tobacco use	No	199	56.7
	Yes	152	43.3
Current alcohol use	No	254	72.4
	Yes	97	27.6
Current chewing khat	No	103	29.3
	Yes	248	70.7
Medication adherence status	Adherent	240	68.4
	Non-adherent	111	31.65
Have admission history	No	201	57.3
	Yes	150	42.7
Have a comorbid physical illness	No	331	94.3
	Yes	20	5.7
Illness duration	< 5 year	141	40.2
	5–9 year	158	45.0
	≥ 10 year	52	14.8
Treatment duration	3- < 6 months	25	7.1
	6 month- 5 year	154	43.9
	≥ 5 year	172	49.0

Other^a^- Tigre, Walayta

### Perceived quality of life of people with schizophrenia

In this study, the respondents’ mean (SD) score of their overall quality of life was 44.81 ± 8.87 and 51.3% (*n* = 180) and 48.7% (*n* = 171) of the study participants scored below the mean and above the mean in overall quality of life respectively. The minimum and maximum mean scores of the study participants in overall quality of life were 19 and 63 respectively. Among the four domains of quality of life, respondents scored highest mean in the physical health domain (12.11 ± 2.04) and scored lowest mean in the social relationship domain (10.14 ± 3.12) respectively (Table 2).

**Table 2:** Mean (SD) scores of quality of life among people with schizophrenia (*n* = 351)

Domains	Mean (± SD)	Range	
		Minimum	Maximum
Physical Health	12.11 ± 2.0	4	19
Psychological Health	11.14 ± 2.26	5	17
Social Relationships	10.14 ± 3.12	4	20
Environmental Health	11.42 ± 2.60	5	17
Overall Quality of life	44.81 ± 8.87	19	63

### Quality of life and its association with current substance use, medication non-adherence and clinical factors of people with schizophrenia

We performed simple and multiple linear regression analyses to identify the association of quality of life with current substance use, medication non-adherence and clinical factors of people with schizophrenia. Quality of life in physical health domain was negatively significantly associated with medication non-adherence (beta = − 4.42, *P* < 0.001), who had comorbid physical illness (beta = − 2.74, *P* < 0.001), current tobacco use (beta = − 1.16, *P* < 0.004) and current chewing khat (beta = − 1.15, *P* < 0.003) and the variance of quality of life in physical health domain was explained 40.6% by all predictors (R^2^ = 0.406, *P* < 0.001). Quality of life in psychological health domain was negatively significantly associated with medication non-adherence (beta = − 4.49, *P* < 0.001), treatment duration (beta = − 0.17, *P* < 0.04), who had comorbid physical illness (beta = − 2.13, *P* < 0.004), current tobacco use (beta = − 1.23, *P* < 0.001) and current chewing khat (beta = − 1.58, *P* < 0.001) and all predictors explain 47.8% of the variance in the psychological health domain of quality of life (Adjusted R2 = 0.478, *P* < 0.001). The social relationships domain of quality of life was negatively significantly associated with medication non-adherence (beta = − 2.29, *P* < 0.001), treatment duration (beta = − 0.14, *P* < 0.005), who had comorbid physical illness (beta = − 1.25, *P* < 0.007) and current tobacco use (beta = − 0.88, *P* < 0.001), and the predictors explain 39.6% of the variance in the social relationships of quality of life (Adjusted R2 = 0.396, *P* < 0.001). The environmental health domain of quality of life was negatively significantly associated with medication non-adherence (beta = − 4.95, *P* < 0.001), treatment duration (beta = − 0.24, *P* < 0.02), who had comorbid physical illness (beta = − 3.39, P < 0.001), current tobacco use (beta = − 1.98, P < 0.001) and current chewing khat (beta = − 2.63, P < 0.001), and the predictors explain 48.1% of the variance in the environmental health domain of quality of life (Adjusted R^2^ = 0.481, *P* < 0.001).

In this study, the overall quality of life has significant negative association with medication non-adherance (beta = − 18.1, *p* < 0.001), treatment duration (beta = − 0.67, *p* < 0.02), having comorbid physical illness (beta = − 9.9, p < 0.001), current tobacco use (beta = − 5.73, *P* < 0.001) and current chewing khat (beta = − 6.22, P < 0.001) and the predictors explained 53.3% of the variance in the overall quality of life (Adjusted R^2^ = 0.533, P < 0.001) (Table 3).

**Table 3:** Multiple linear regression analysis of quality of life with medication non-adherence, current substance use and clinical factors of people with schizophrenia (*n* = 351)

	Physical Health		Psychological Health		Social Relationships		Environmental Health		Overall quality of life	
*Variables*	***Beta***	***P***	***Beta***	***P***	***Beta***	***P***	***Beta***	***P***	***Beta***	***P***
*Medication non-adherence*	−4.42	0.001	−4.49	0.001	−2.29	0.001	−4.95	0.001	−18.1	0.001
*Illness duration*	0.01	0.87	0.03	0.75	0.07	0.15	0.07	0.49	0.15	0.6
*Rx duration*	−0.13	0.12	−0.17	0.04	−0.14	0.005	− 0.24	0.02	−0.67	0.02
*Have admission history*	−0.46	0.22	−0.41	0.24	−0.11	0.62	−0.60	0.18	−1.8	0.15
*Have a comorbid physical illness*	−2.74	0.001	− 2.13	0.004	−1.25	0.007	−3.39	0.001	−9.9	0.001
*Current tobacco use*	−1.16	0.004	−1.23	0.001	−0.88	0.001	−1.98	0.001	−5.73	0.001
*Current chewing khat*	−1.15	0.003	−1.58	0.001	−0.39	0.10	−2.63	0.001	−6.22	0.001
*Current alcohol use*	−0.12	0.75	0.52	0.09	−0.26	0.27	0.71	0.14	0.74	0.55

## Discussion

The current study aimed to investigate the quality of life and its association with medication non-adherence, current substance use and clinical factors of people with schizophrenia attending follow up service at Jimma University Medical Center, psychiatry clinic, Southwest Ethiopia. This study was the first study in Ethiopia to investigate the association of medication non-adherence and substance use with the quality of life of people with schizophrenia. In this study, the participants’ mean score of overall quality of life was found to be (44.81 ± 8.87) and the domain with the lowest mean score was the social relationships domain (10.14 ± 3.12).

In our study, the 111(31.65%) of participants were non-adherent to their medication and this result was higher than study conducted at Ayder Referral Hospital, Mekelle, Northern Ethiopia which revealed 26.5% of patients with schizophrenia were non-adherent to their antipsychotic medication [29] and the result was lower than finding of the study conducted at Bahir Dar Felege Hiwote Referral hospital, northwest Ethiopia which revealed overall prevalence of medication non-adherence was 55.2% [30] This difference could be due to the different tools used to measure medication non-adherence. The lowest score on the social relationship domain of QoL among people with schizophrenia in our study could be due to the negative symptoms, which affect the patient’s ability to live independently, to perform activities of daily living, to be socially active and maintain personal relationships. Also, it could be due to the stigma towards mental illness, which excludes the mentally ill from social life (21).

In the current study, medication non-adherence was significantly and negatively associated with all domains of quality of life and with the overall quality of life, and this result was in line with the previous study [20]. This finding indicates that medication adherence plays a key role in the quality of life of people with schizophrenia and improving medication adherence is a good approach to improve the quality of life of people with schizophrenia.

In our study, regarding the association between quality of life and clinical factors, treatment duration has a significant negative association with psychological domain, social relationships, environmental domain and overall quality of life. The possible justification behind this finding will be as reported in the previous study, treatment duration and illness duration have a significant association with medication non-adherence among people with schizophrenia [31] and as explained in the current study, medication non-adherence has a significant negative association with participants’ quality of life. Because of this, the treatment duration of people with schizophrenia could affect their quality of life by affecting medication adherence. In our study also, having the comorbid physical illness was significantly negatively associated with a physical domain, psychological domain, social relationships, environmental health domain and overall quality of life and this result is supported by the previous study which revealed people with schizophrenia alone had a better QoL than those with any multimorbidity [32]. This finding indicates that early detection and treatment of comorbid physical illness among people with schizophrenia will have significant importance to improve their overall quality of life.

This study result also revealed current tobacco use has significant negative association with all domains of quality of life including physical health, psychological health, social relationships, and environmental health domains as well as with overall quality of life and the current chewing khat has significant negative association with physical, psychological, environmental domains and overall quality of life. This finding was in line with the previous study results which revealed that schizophrenic patients with substance use disorder comorbidity would report poorer QoL scores than non-comorbid patients [17, 33]. There are different possible justifications why patients with schizophrenia and comorbid substance use have poorer QoL scores. These justifications include: Patients with comorbid substance use would have poor interpersonal relationships than non-substance-using patients. Because these patients could spend their time searching and using substances [1]. Again substance use, especially during either intoxication or withdrawal time, could cause behavioral change and even it could directly worse psychiatric symptoms and could affect their psychological health besides social relationships. Substance use can also affect their coping skills with illness by making them non-compliance to the treatments [1].

Our study was not without limitations. The WHOQoL-BREF scale of Quality of Life measures only capture of a small point of time (in the last 2 weeks) in the patients’ life and it is difficult to generalize to patients overall QoL. Due to its cross-sectional nature, the study could not explore the cause and effect relationships of variables. Questions about substance use are sensitive by nature and there might be underreporting of using substances.

## Conclusions

In our study, the social relationship domain of quality of life of people with schizophrenia has the lowest mean score. Medication non-adherence, current tobacco use, current chewing khat, treatment duration, and having comorbid physical illness were found to have a statistically significant association with the overall quality of life of people with schizophrenia. This study recommends that all stakeholders including the health professionals who are treating people with schizophrenia should have to give priority for interventions that improve their social deficits and medication adherence, screen and treat for their comorbid physical illness and decreasing substance abuse is imperative.

## Data Availability

The datasets used and/or analyzed during the current study are available from the corresponding author on reasonable request by tilahunabdeta@gmail.com

## Abbreviations

DSM-IV/V: Diagnostic and Statistical Manual of Mental Disorders, Version IV/V
JUMC: Jimma University Medical Center
OPD: Outpatient Department
QoL: Quality of Life
WHO: World Health Organization
WHOQoL-BREF: World Health Organization Quality of Life Assessment Short version
